# Development of a Nuclear Transformation System for Oleaginous Green Alga *Lobosphaera (Parietochloris) incisa* and Genetic Complementation of a Mutant Strain, Deficient in Arachidonic Acid Biosynthesis

**DOI:** 10.1371/journal.pone.0105223

**Published:** 2014-08-18

**Authors:** Boris Zorin, Omer Grundman, Inna Khozin-Goldberg, Stefan Leu, Michal Shapira, Yuval Kaye, Nicolas Tourasse, Olivier Vallon, Sammy Boussiba

**Affiliations:** 1 Microalgal Biotechnology Laboratory, French Associates Institute for Agriculture and Biotechnology of Drylands, J. Blaustein Institutes for Desert Research, Ben-Gurion University of the Negev, Midreshet Ben-Gurion, Israel; 2 Department of Life Sciences, Ben-Gurion University of the Negev, Beer Sheva, Israel; 3 UMR 7141 CNRS/Université Pierre et Marie Curie, Institut de Biologie Physico-Chimique, Paris, France; University Paris South, France

## Abstract

Microalgae are considered a promising source for various high value products, such as carotenoids, ω-3 and ω-6 polyunsaturated fatty acids (PUFA). The unicellular green alga *Lobosphaera (Parietochloris) incisa* is an outstanding candidate for the efficient phototrophic production of arachidonic acid (AA), an essential ω-6 PUFA for infant brain development and a widely used ingredient in the baby formula industry. Although phototrophic production of such algal products has not yet been established, estimated costs are considered to be 2–5 times higher than competing heterotrophic production costs. This alga accumulates unprecedented amounts of AA within triacylglycerols and the molecular pathway of AA biosynthesis in *L. incisa* has been previously elucidated. Thus, progress in transformation and metabolic engineering of this high value alga could be exploited for increasing the efficient production of AA at competitive prices. We describe here the first successful transformation of *L. incisa* using the *ble* gene as a selection marker, under the control of the endogenous *RBCS* promoter. Furthermore, we have succeeded in the functional complementation of the *L. incisa* mutant strain P127, containing a mutated, inactive version of the delta-5 (Δ5) fatty acid desaturase gene. A copy of the functional Δ5 desaturase gene, linked to the *ble* selection marker, was transformed into the P127 mutant. The resulting transformants selected for zeocine resistant, had AA biosynthesis partially restored, indicating the functional complementation of the mutant strain with the wild-type gene. The results of this study present a platform for the successful genetic engineering of *L. incisa* and its long-chain PUFA metabolism.

## Introduction

The green freshwater microalga *Lobosphaera incisa* (Reisigl) comb. nov. was isolated from snow water patches in the alpine environment of Mt. Tateyama, Japan [1]. It belongs to the Trebouxiophyceae, a class of Chlorophyte algae. *Lobosphaera incisa* was initially assigned to the genus *Parietochloris* rather than to *Myrmecia* [1] but was recently reclassified as belonging to *Lobosphaera* based on zoospore morphology and 18S rDNA analysis [2]. In contrast to most algae whose storage lipids triacylglycerols (TAGs) are mainly composed of saturated and monounsaturated fatty acids (FA), the main fatty acid in TAGs of *L. incisa* is arachidonic acid (AA, 20∶4 *n*-6). When cultivated under nitrogen starvation, the total FA content of the alga is over 35% of dry weight. AA constitutes about 60% of total FAs (TFA), and over 90% of cell AA is deposited in TAGs, making *L. incisa* the richest plant source of the pharmaceutically and nutraceutically valuable AA and a target organism of high biotechnological interest [3] [4].

The ability to store AA within reserve lipids, TAGs, was thought to be related to the need to maintain a buffering capacity for the long-chain polyunsaturated FA (LC-PUFA) pool under conditions of temperature change or growth recovery when rapid LC-PUFA incorporation into the cellular membranes is required for adjusting membrane fluidity [5] [6]. The conversion of dihomo-γ-linolenic acid (DGLA, 20∶3 *n*-6) to AA is mediated by the enzyme Δ5 desaturase (*DES5*, GenBank: GU390533). Based on its chlorotic phenotype at 15°C, we have been able to isolate P127, a *L. incisa* mutant strain in *DES5* [7] [8]. The chemically induced non-sense mutation caused a nucleotide alteration in the *DES5* gene, leading to the appearance of stop codon and the complete loss of AA biosynthesis. P127 accumulates DGLA, instead of AA, in TAGs and in polar lipids [8] [9].

In order to improve the biotechnological potential of *L. incisa*, we developed a stable nuclear transformation system for its metabolic engineering. Our initial attempts to transform this alga used the *Streptomyces hindustanus ble* gene as a selection marker connected to heterologous promoters, such as popular for transformation of *Chlamydomonas reinhardtii* tandem *HSP70A/RBCS2* promoter [10], or the viral CaMV 35S. Using such constructs, transformation both by biolistic delivery and electroporation failed. Recent studies in microalgae engineering indicate that the best transformation results are achieved by using cloned endogenous promoters and regulatory untranslated regions (UTRs), fused to suitable selection markers. In *C. reinhardtii*, the *PSAD*, *RBCS2* and *HSP70A* promoters have been widely used to drive nuclear gene expression [11]–[13]. Other endogenous promoters have been used to drive the expression of the *ble* gene, which encodes a protein that confers resistance to the antibiotic zeocin in many algal species. For example, the *fcp* promoter was successfully used for transformation of *Phaeodactylum tricornutum* [14] and likewise the bidirectional *VCP2* promoter for transformation of *Nannochloropsis sp*. [15].

Here we describe the development and characterization of a transformation vector for the genetic engineering of *L. incisa*, based on the endogenous Ribulose-1,5-bisphosphate carboxylase/oxygenase (RuBisCO) small subunit (*RBCS*) promoter for expressing the *ble* gene as a selection marker. In order to demonstrate the successful metabolic engineering of *L. incisa*, we also transformed the P127 mutant, using the *ble* marker linked to the wild-type genomic *DES5* and demonstrate successful restoration of AA biosynthesis.

## Materials and Methods

### Algal strains and culture conditions

We used the original isolate of *L. incisa* [1], maintained in the Microlagal Biotechnology Laboratory, Ben Gurion University, Israel, and now deposited at the Culture Collection of Algae at Göttingen University under accession no. SAG 2468. Axenic cultures were cultivated mixotrophically in LB-Miller Broth medium (BD, Franklin Lakes, NJ), either in liquid or on solid agar (Difco) medium. For *C. reinhardtii*, we used the cell-wall-deficient expression strain UVM4 [16], kindly provided by R. Bock, cultivated mixotrophically in a Tris-Acetate-Phosphate (TAP) medium [17]. All strains were cultivated in 250-mL Erlenmeyer flasks in an incubator shaker at a speed of 170 rpm, under an air/CO_2_ atmosphere (99∶1, v/v), at 25°C and an illumination of 100 µmol photons m^−2^ s^−1^ PAR.

### DNA isolation

Genomic DNA (gDNA) was extracted from algal cells following the modified cetyltrimethyl ammonium bromide (CTAB) based protocol. CTAB buffer (2% CTAB, 1.4M NaCl, 0.2% β-mercaptanol, 20 mM EDTA, 100 mM Tris-HCl, pH 8.0) preheated to 60°C was added to cell pellets ground in liquid nitrogen. After incubation at 60°C in the water bath for 30 minutes, samples were extracted twice by chloroform-isoamyl alcohol (24∶1). Cold isopropanol was added to the upper aqueous phase, and the DNA was precipitated by centrifugation. The pellet was washed in 70% ethanol and resuspended in PCR grade water. The concentration and purity of the DNA was determined by Nano-Drop (Thermo-Scientific, Logan, UT).

### Isolation of RNA and cDNA synthesis

The cells from 10 mL of culture with the chlorophyll content of 30 mg L^-1^ were harvested by centrifugation at 4000 rpm for 5 min, washed with double distilled sterilized water, flash-frozen in liquid N, and stored at −80°C until further use. Total RNA was isolated following the SV Total RNA Isolation system protocol (Promega, Madison, WI). cDNA was prepared from 1 µg of total RNA-template with the Verso cDNA kit (Thermo Fisher Scientific, Epson, UK).

### Cloning procedures

PCR products were generated using the AccuPower PCR PreMix (Bioneer, Alameda, CA) and a TProfessional Thermocycler (Biometra, Goettingen, Germany). All PCR reactions were made on standard conditions. The sequences of all primers used are given in Table 1. PCR products were purified from agarose gel using the AccuPrep Gel Purification Kit (Bioneer). The In-Fusion HD Cloning Kit (Clontech, Mountain View, CA) was used for the directional cloning of DNA fragments into target vectors, according to the manufacturer's instructions.

**Table 1:** Primers used in this study.

Primer	Sequence
RbcS1kbF	5′-GAGTCACGACGGACTGTAGGA-3′
RbcSR	5′-TGCTTGGTAGCACAGTTCAGA-3′
D5DF	5′-AGTCTTGTACTCCTTGCCCTCCT-3′
D5DR	5′-GCTCTGTAAATCCATCCATCGTC-3′
RbcEmptyF	5′-TAAGCAACGAATGGCCTAG-3′
RbcEmptyR	5′-CATCTCGACTTGTTTAGTGTTG-3′
BleF	5′-ACACTAAACAAGTCGAGATGGCCAAGCTGACCAGCGCCGT-3′
BleR	5′-CCTAGGCCATTCGTTGCTTAGTCCTGCTCCTCGGCCACGAA-3′
RbcS450F	5′-GTTGATAGGCAAGACCCAACA-3′
RbcS200F	5′-GTTGCTTCACTTTCGCTTGAC-3′
RT_LiDes5 F	5′-TAAGTGCCAGGGCTGTGCTAGA-3′
RT_LiDes5 R	5′-GAACTGACCCTCCTCTGTGTCCT-3′
RT_LiAct F	5′-CGTCCAGCTCCACGATTGAGAAGA-3′
RT_LiAct R	5′-ATGGAGTTGAAGGCGGTCTCGT-3′
LiDes5 F3	5′-GCTGTAACAGAGGGCGCTG-3′

### Cloning of RBCS and DES5 genes

The *RBCS* gene, including 1 kb upstream of the predicted start codon and 430 bp downstream of the stop codon, was amplified using primers that annealed to the 5′ (RbcS1kbF) and 3′ (RbcSR) ends of the gene sequence. The 6048 bp genomic loci of *L. incisa* containing *DES5* gene, including 1.8 kb upstream of the predicted start codon and 1 kb downstream of the stop codon, was amplified using primers that annealed to the 5′ (D5DF) and 3′ (D5DR) ends of the gene sequence. The amplification was carried out by PCR from 5 ng of total *L. incisa* gDNA as described above. The *RBCS* gene was cloned into the pGEM-T easy vector (Promega, Madison, WI) to create pLiRbcS plasmid. The *DES5* gene was cloned into the pJET1.2 vector (Thermo-Scientific) to create pJET-LiD5Des plasmid.

### Transformation vector design

**Figure 1:**
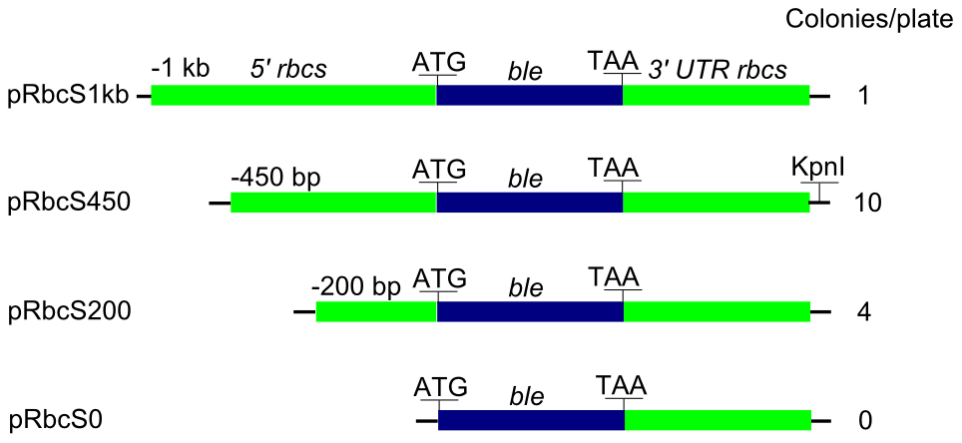
Schematics of the *LiRbcS* transformation constructs and corresponding average number of resultant colonies per plate. The number of colonies is the average of 12 different transformation events. The blue boxes represent the *ble* coding sequence, and the green boxes represent the *LiRbcS* promoter and terminating sequence. *KpnI* restriction site used in Southern blot hybridization is marked on the construct that was used for *L. incisa* transformation.

**Figure 2:**
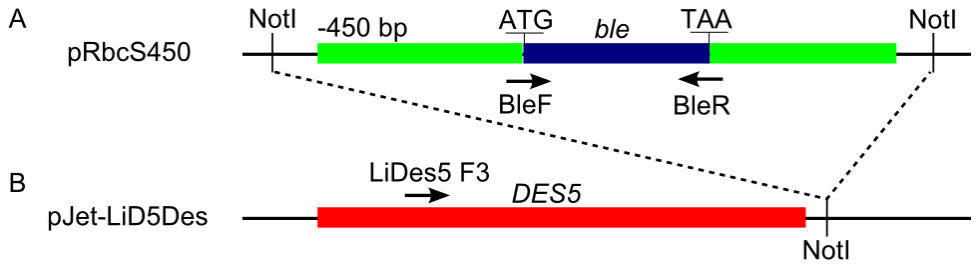
Schematic diagram showing the construction strategy of pD5Dselect. The pRbcS450 plasmid (A) contains the selection cassette, flanked by *NotI* restriction sites. The pJET-LiD5Des plasmid (B) contains the functional *DES5* gene with its flanking regions. Following *NotI* digestion, the selection cassette was subcloned into pJET-LiD5Des. Primers used in analysis of P127-transformant are marked.

The CDS of the *ble* selection marker was amplified from the plasmid pGenD-Ble [11] with the primers BleF and BleR. Using the In-Fusion protocol, it was cloned into pLiRbcS in replacement of the *RBCS* CDS, yielding the initial transformation vector pRbcS1kb. For the following cloning step, pRbcS1kb was used as a template for the amplification of expression cassettes with different promoter lengths. The forward primers RbcS450F, RbcS200F and BleF were used, in conjunction with the reverse primer RbcSR, to amplify the expression cassette with promoter lengths of 450 bp and 200 bp and without promoter, respectively. The PCR products were cloned into the pGEM-T easy vector to obtain three additional transformation vectors: pRbcS450, pRbcS200 and pRbcS0 (Fig. 1). Later, the pRbcS450 selection cassette was subcloned into pJET-LiD5Des by flanking *Not*I restriction sites to create the pD5Dselect cassette (Fig. 2). Plasmid midipreps of the constructs were prepared using the Fast Ion Midiprep Kit (RBC Bioscience, New Taipei City, Taiwan). The concentrations of the plasmids were determined by spectrophotometric analysis, and 1 µg/µL aliquots of DNA were prepared for later transformations.

### Transformation of *C. reinhardtii*

Cells of *C. reinhardtii* UVM4 in the log phase were transformed according to the PEG-glass beads method [18]. Prior to use, the vectors were linearized by *Sca*I. Cells were poured onto selective TAP agar plates (20 µg mL^−1^ zeocin) and incubated at 25°C under a light intensity of 20 µM photons m^−2^ s^−1^. Transformants appeared after 2–3 weeks of incubation.

### Transformation of *L. incisa* by electroporation

Electroporation was performed using a Bio-Rad Gene PulserXcell as described by [19]. *L. incisa* was grown in 100 mL LB to a chlorophyll content of 30 µg mL^−1^. Then 20 mL of cell culture in 50 mL conical centrifuge tube were placed on ice, sonicated for 30 seconds at 50% power using a Digital Sonifier Cell Disruptor (Branson Ultrasonics Corp., Danbury, CT), centrifuged. The cell pellet was washed twice with sterile water by sequential centrifugation at 3000 rpm at ambient temperature. Prior to transformation by electroporation, the sample was observed under light microscope to ensure sufficient cell clusters disintegration. The pellet was resuspended in 0.4 mL of sterile water followed by addition of 10 µg of linearized plasmid DNA, and then transferred into a 2 mm electroporation cuvette for electroporation [one 500 V pulse (at 2500 V/cm), 50 mF capacity and infinitive shunting resistance]. Transformed cells were transferred to Erlenmeyer flasks containing 20 mL of LB medium and incubated for 24 h in the dark, followed by 24 h under light intensity of 20 µM photons m^−2^ s^−1^ in ambient air condition with moderate agitation. Afterwards, transformed cells were plated onto selective LB-agar plates, containing 10 µg mL^−1^ zeocin at 25°C under a light intensity of 20 µM photons m^−2^ s^−1^.

### Southern blot analysis

Southern blot hybridization was performed using the North2South Chemiluminescent Hybridization and Detection Kit (Thermo Scientific) according to the manufacturer's instructions. Briefly, *L. incisa* genomic DNA (5 µg) was digested with *KpnI* endonuclease (NEB), desalinated by EtOH precipitation and separated by electrophoresis on a 0.7% agarose (Seakem, Lonza) gel. After depurination with 250 mM HCl, incubation in denaturation solution (0.5 M NaOH, 1.5 M NaCl) and neutralization with 0.5 M Tris-HCl pH 7.5, 1.5 M NaCl, the gel was equilibrated with 20× SSC (saline-sodium citrate buffer, 3 M sodium chloride and 300 mM trisodium citrate, pH 7.0). After overnight capillary transfer of digested and separated gDNA from the gel to a Magnagraph nylon membrane (GE Water & Process Technologies, USA) in 20× SSC buffer, the membrane was rinsed with 2× SSC and baked for 2 h at 80°C. The membrane was blocked, hybridized and washed, and bands were detected according to the kit manual. Chemiluminescent detection was performed using a Microchemi camera (Bioimaging System, Israel). The probe was synthesized by PCR using primers BleF and BleR (Table 1) from pRbcS450 plasmid with a GoTaq (Promega) PCR mix using the plasmid DNA as the template and biotinylated dUTP (Thermo Scientific) added to a final concentration of 50 nM. Efficiency of probe-labeling was verified by electrophoresis on an agarose gel and comparing molecular weights of labeled and unlabeled PCR products; the amount of probe was quantified using a ND-1000 spectrophotometer (NanoDrop).

### Quantitative real-time PCR (qRT-PCR)

qRT-PCR analysis was carried out in optical 96-well plates using the CFX96 Touch Real-Time PCR Detection System (Bio-Rad, Hercules, CA). qRT-PCR primer pairs (Table 1) were designed for *LiDES5* and the housekeeping gene *LiAct* (actin) and the reactions were performed using iTaq Universal SYBR Green Supermix (Bio-Rad). PCR cycling conditions consisted of an initial polymerase activation step at 95°C for 30 s followed by 40 cycles at 95°C for 5 s and 60°C for 30 s, and a final melting step at 65–95°C. Results were analyzed using the 2^−ΔΔCt^ method, a function of the CFX Manager Software v3.0, using the relative expression value of the housekeeping gene as the calibrator. The experiment was performed twice, with two biological replicates and three technical replicates for each sample.

### Fatty acid analysis

The direct transmethylation of algal biomass was performed by incubating freeze-dried biomass in dry methanol containing 2% (v/v) H_2_SO_4_ at 80°C for 1.5 h under argon atmosphere with continuous stirring as previously described [20]. Heptadecanoic acid (C17∶0) (Fluka, Buchs, Switzerland) was added as an internal standard. FAME were quantified on a Trace GC Ultra (Thermo, Milan, Italy) equipped with a flame ionization detector (FID) and a programmed temperature vaporizing (PTV) injector. The detector temperature was fixed at 280°C, and helium was used as a carrier gas. The PTV injector was programmed to increase the temperature from 40°C at time of injection to 300°C at time of sample transfer. Separation was achieved on fused silica capillary columns (SUPELCOWAX 10, Sigma-Aldrich, 30 m×0.32 mm). FAME were identified by co-chromatography with authentic standards (Sigma-Aldrich).

## Results

### Cloning and analysis of the *L. incisa RBCS* and *DES5* genes

The *RBCS* and *DES5* genes, their putative promoters and 3′-flanking flanking regions were identified in a draft assembly of the *L. incisa* genome generated in the framework of the EU FP7 GIAVAP project (www.GIAVAP.eu). The sequences were submitted to Genbank, accession numbers KJ633120 and KJ633121 for RBCS and DES5 genes respectively. The *RBCS* gene contains two introns within the 570 bp coding sequence, while *DES5* contains seven introns within the 1356 bp of CDS. Genes and their upstream and downstream sequences were cloned into plasmid vectors.

### Characterization of *L. incisa RBCS* promoter and UTRs in *C. reinhardtii*

The *RBCS* based transformation vectors (Fig. 1) were used to transform *C. reinhardtii* cells. After 2–3 weeks on zeocin selection plates, resistant colonies appeared. For all transformants, the presence of the *ble* gene was confirmed by PCR (not shown). Each construct was used for 12 different transformation events. The highest number of colonies was recovered with the pRbcS450 construct with an average of 10 colonies per plate. Four colonies per plate, on average, were recovered using pRbcS200, but none ever with pRbcS0, indicating that the *RBCS* promoter was indeed necessary to drive *ble* expression. Constructs with the 1 kb promoter sequence resulted in a very low transformation efficiency, suggesting that the −1000 to −450 region of the *L. incisa RBCS* promoter contains elements counteracting transformation or gene expression in *C. reinhardtii*.

### Genetic transformation of *L. incisa*

**Figure 3:**
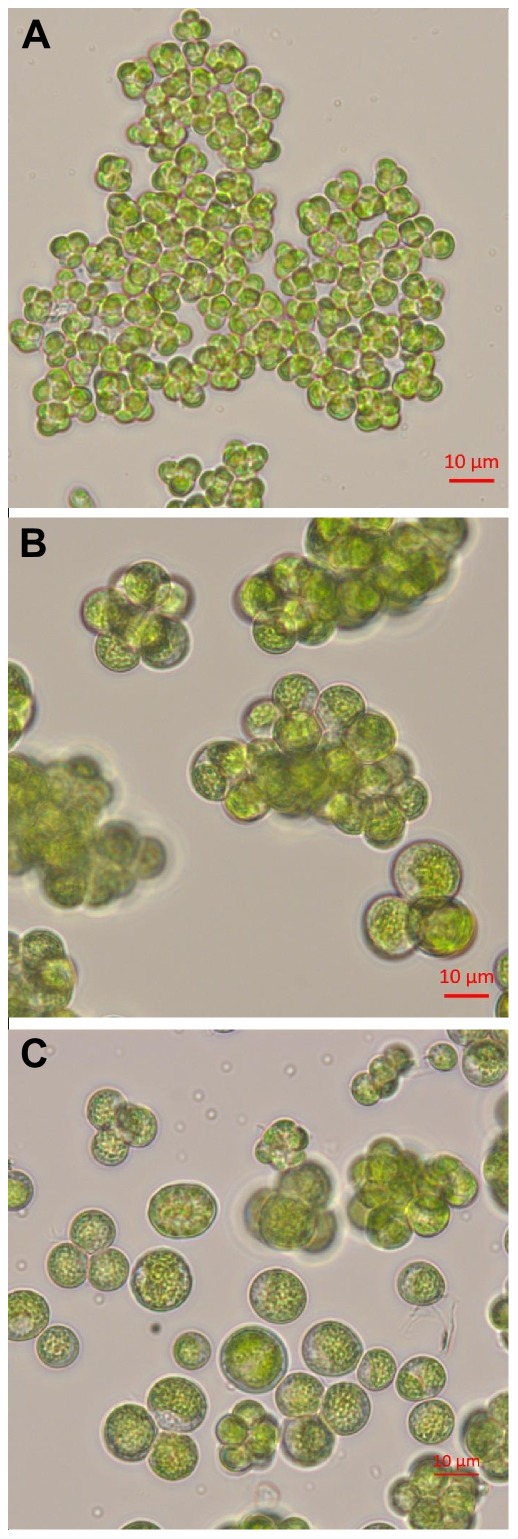
The effect of growth media and sonication on the morphology of *L. incisa* cells. (A) cells grown in a mineral BG11 medium, (B) cells grown in an LB medium, (C) cells grown in an LB medium, after sonication.

*L. incisa* is usually cultivated and maintained in the photoautotrophic mineral medium BG11 [21]. In this medium, *L. incisa* divides through the multiple fission of mother cells, producing 8–16 daughter cells after hatching. After release, daughter cells primarily remain attached to each other, giving rise to large cell clusters (palmelloids) (Fig. 3a). We assume that our previous failures in transformation attempts were due to difficulties in delivering plasmid-DNA into a single cell. Indeed, while cells remain in clusters, many dividing cells are shielded by sister-cells that might prevent plasmid penetration. Moreover, we assume that even if one cell from a cluster obtains and inserts into its genome a copy of plasmid DNA, the chances for survival of the single transformed cell during selection would also be low, because of the proximity of dying cells, which might release toxic and cell-death signaling compounds. Hence, our first aim for achieving a successful *L. incisa* transformation was the production of a culture enriched in single cells.

In the course of our studies on *L. incisa*, we have determined that this alga can grow well mixotrophically in LB medium. We have observed that in these cultures, *L. incisa* seems to form smaller clusters that further disassemble into single cells, even under the weight of a microscopic cover-glass (Fig. 3b). We assumed that medium enrichment with the organic carbon and nitrogen sources, such as sugars, amino acids and peptides, that are abundant in LB medium, might have exerted a substantial impact on the cell cycle and/or cell wall composition, resulting in the reduced attachment of cells and clusters formation. We have hypothesized that the single-celled form of the culture might be produced from cells growing in the LB medium after gentle mechanical treatment. Thus, in order to develop a stable transformation system, we identified sonication with precise power and time settings as the most suitable treatment for preparing high quality single-cell-enriched cultures for transformation (Fig. 3c). Since counting of individual cells was difficult even after ultrasonication, due to some remaining clusters, we estimated transformation efficiency not per number of cells in transformation reaction but per standard reaction of 20 mL containing 30 µg mL^−1^ chlorophyll and 10 µg of linear plasmid DNA. The transformation efficiency in optimized conditions was approximately 10 clones per 10 µg of plasmid DNA for wild-type *L. incisa* and a magnitude lower for the P127 mutant strain.

### Confirmation of stable DNA integration to the *L. incisa* genome

**Figure 4:**
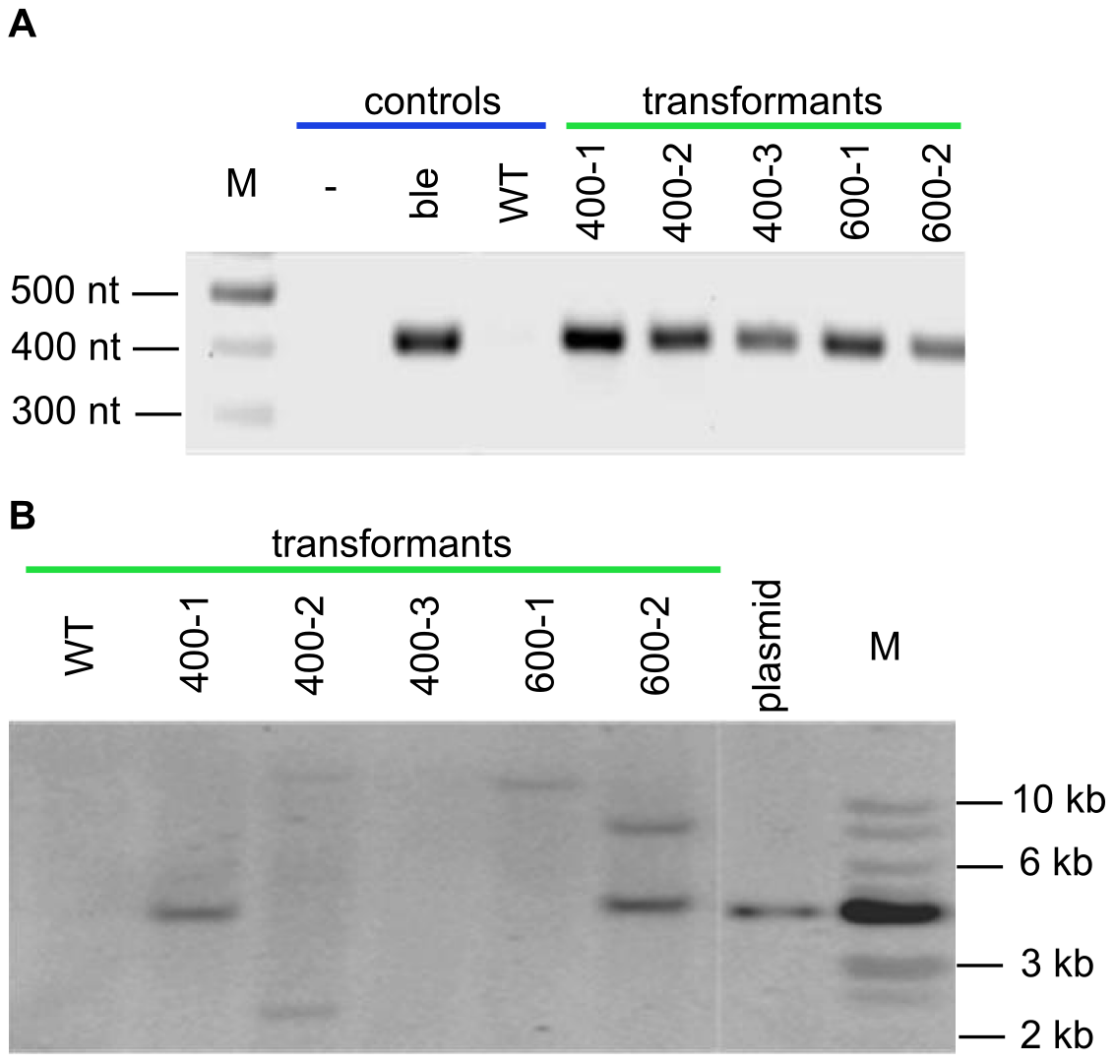
Molecular analyses of *L. incisa* clones transformed with a pRbcS450 construct. (A) PCR analysis: gDNA was amplified with BleF and BleR primers yielding a 415-bp fragment. Lanes: (M) DNA ladder; (-) no template, (Ble) *ble* plasmid control, (wt) negative control (non-transformed cells); five transformed clones. (B) Southern blot analysis: gDNA isolated from both transgenic and non-transgenic cells, as well as plasmid DNA (pRbcS450), were digested with *KpnI* restriction enzyme. The blot was hybridized with a probe derived from a 415-bp amplified fragment of the *ble* gene. Lanes: (M) 1 Kb ladder; (plasmid) positive control; five transformed clones; (wt) negative control (non-transformed cells).

Zeocin resistant colonies were transferred to new selective media, and the integration of the transgene was confirmed by PCR and Southern blot analysis. The PCR analysis was carried out on five zeocin-resistant clones, obtained after transformation with plasmid pRbcS450. The expected 415 bp *ble* fragment was detected in all five clones but not in non-transformed cells (Fig. 4A). Southern blot analysis was performed to confirm the stable integration of the transgene (*ble*), as well as to estimate the number of copies of the transgene inserted into the genome of transgenic clones. The gDNAs of the five transformants and wild-type were digested with *Kpn*I restriction enzyme and analyzed by hybridization with a *ble*-specific probe (Fig. 4B). Since pRbcS450 plasmid was digested only once with *KpnI* restriction enzyme at the 3′ flanking region of *ble* gene-marker, we expected that size of hybridized bands would be unique for each integration event since a second 5′ *KpnI* restriction site should be randomly located at the 5′ chromosome flanking region. According to the hybridization pattern, two out of the five lines exhibited two hybridization bands (400-2 and 600-2), while two lines (400-1 and 600-1) exhibited only one. Clone 400-3 did not exhibit any hybridization signal, probably due to poor DNA quality. No bands were observed in the wild-type non-transformed cells, and a single sharp band was observed in the digested plasmid DNA used as a positive control.

**Figure 5:**
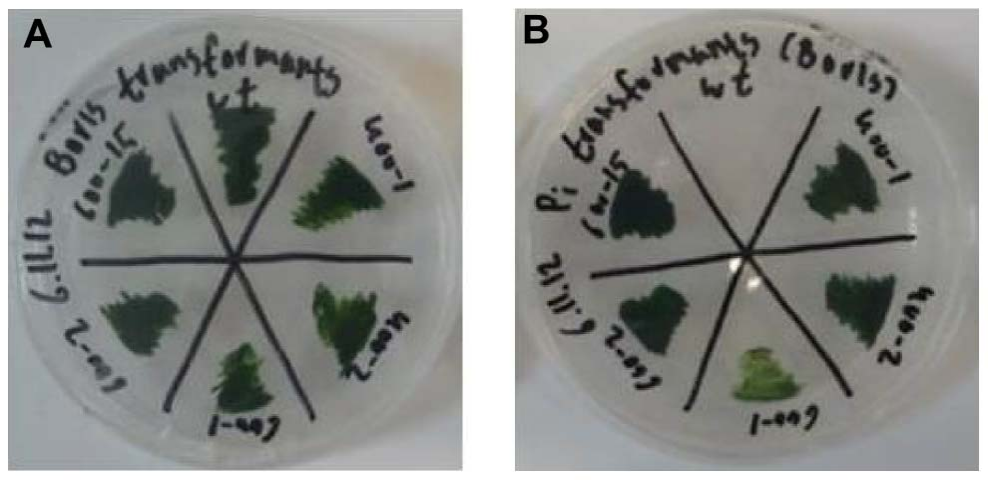
Transformation stability analysis. (A) five resistant clones and a wild-type control were grown on non-selective plates for three months. The cell-lines were then restreaked on a selective plate. (B) all clones retained their antibiotic resistance. The wild-type did not grow under selection (20 µg mL^−1^ zeocin).

Transformation stability was assessed by growing the resistant clones on non-selective plates for three months and then, for an additional month, on selective plates (Fig. 5). All clones retained their antibiotic resistance.

### Complementation of the mutant P127 with functional DES5

**Figure 6:**
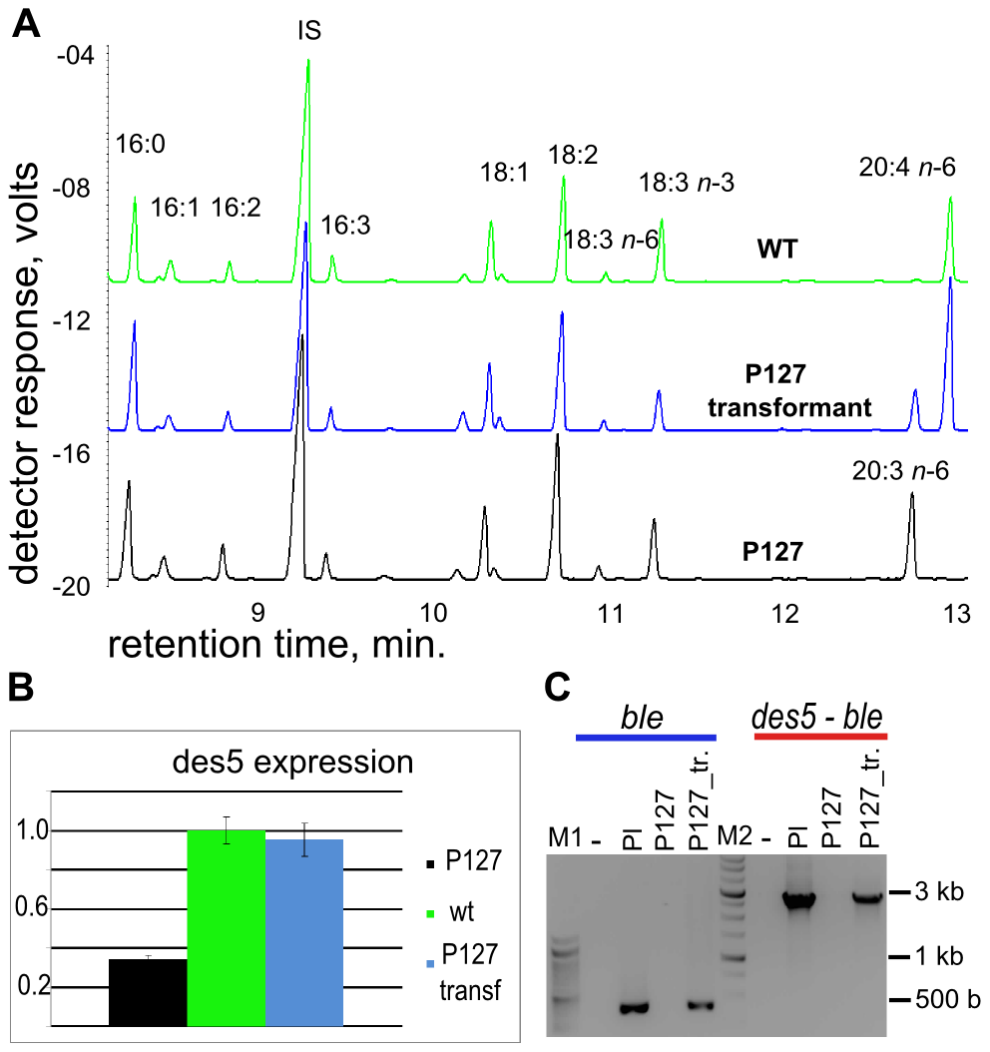
Analysis of *L. incisa* P127 clones transformed with a pD5Dselect construct. (A) GC-FID analysis of the fatty acid profiles of P127 mutant, P127-transformant and wild type cells, (B) qRT-PCR analysis of expression of *DES5* gene in P127 mutant, P127-transformant and wild type cells. (C) PCR analysis of gDNA isolated from P127 mutant, P127-transformant and wild type cells with primers *Li*Des5 F3 and BleR, demonstrating transgene integration in conjunction with the *ble* gene.

Having established the transformation platform, we attempted to restore AA biosynthesis in the previously isolated *des5* mutant strain P127 [9] by metabolic engineering. The linearized plasmid pD5Dselect harboring the genomic version of *DES5* was introduced into P127 cells by electroporation, as described above. Notably, the transformation efficiency, even with pRbcS450 plasmid, of the P127 mutant appeared to be substantially lower than that of the wild-type. Zeocin resistant colonies appeared after four weeks. Insertion of the pD5Dselect cassette containing wt-*DES5* gene linked to *ble* gene marker was confirmed by PCR amplification from gDNA isolated from P127-transformants. We used BleF and BleR primers for *ble* gene amplification and *Li*Des5 F3 and BleR primers (Table 1) for conformation of a linkage *DES5* and *ble* genes in the genome of P127-transformants (Fig. 6C).

The P127 mutant strain, the P127-transformant and the wild type strain were cultivated in mBG-11 liquid medium and harvested for FA analysis. A gas chromatography (GC) analysis of FA composition, following the growth in the complete mBG-11 nutrient medium with daily dilution for 4 days to keep the logarithmic growth, revealed a drastic decrease in the proportion of DGLA in the fatty acid profile (from *ca*. 16% to 6.3% of TFA) with a concomitant rise in that of AA in the P127-transformant as compared to the untransformed P127 strain. The representative chromatograms are shown on Fig. 6A. Notably, the AA proportion in the FA profile of the recombinant strain (25% of TFA) reached and even surpassed the AA proportion in the WT (16% of TFA). Furthermore, as determined by qPCR, while the level of *DES5* transcript expression was lower in the mutant than in the WT, pD5Dselect integration led to a similar level of expression in the WT and in the P127-transformant (Fig. 6B). However, the P127-transformants that featured relatively high proportion of DGLA simultaneously with the restored AA biosynthesis, appeared to display an altered slow-growth phenotype. The impaired growth of the P127-transformant may be responsible for its higher AA percentage compared to the WT because growth inhibition in this alga is associated with the production of storage lipids that are rich in LC-PUFA. At present, we do not have a plausible explanation for this phenomenon; future studies will shed more light on the effect of altered LC-PUFA content on physiological response of *L. incisa* to cultivation conditions.

## Discussion

Microalgae have a high potential for the production of biofuels and high value products, such as PUFA and carotenoids [22]. However, microalgal production processes require significant improvements at various levels to compete successfully with products currently used in food, cosmetics, aquaculture and agriculture that are prepared synthetically or extracted from other natural feedstock. Significant progress in strain development and sustainable cultivation technologies are required to reduce the currently high production costs for algal biomass that is produced phototrophically. To date, a few green microalgae species such as *Chlamydomonas, Dunaliella* or *Chlorella*, and heterokont microalgae such as *Phaeodactylum* or *Nannochloropsis* have been successfully transformed [23]. Beyond mere transformation, adequate tools for the actual expression of unselected transgenes are required: they have been efficiently demonstrated so far in only a few algal species. Here, we demonstrate the ability: i) to genetically transform *L. incisa* and ii) to use this transformation platform for the metabolic engineering of its FA composition. *L. incisa* is highly attractive alga species, that can accumulate over 20% of its dry-weight (% of DW) as AA under nitrogen starvation [3] [4], but its industrial use is hampered by relatively slow growth and sensitivity to environmental stress, such as combined stress caused by high light and nitrogen depletion. The technology presented here can thus assist in modulating the LC-PUFA composition of this alga through sequential metabolic engineering of FA desaturation level and carbon chain-length. Dramatic increases in the C22 ω-3 LC-PUFA by iterative engineering of the biosynthesis pathway have been achieved in *P. tricornutum* [24]. The developed tools for *L. incisa* pave the way to further engineering efforts to improve growth performance and/or lipid productivity, as exemplified in several microalgae [24]–[26]. Further progress could come, for example, from downregulation of lipid catabolism, which in *Thalassiosira pseudonana* improved lipid yield without affecting growth performance [26], and enhancement of photosynthesis by modulating inorganic carbon assimilation (Spalding MH. personal communication). An expected doubling of productivity would allow the use of this phototrophic organism for competitive PUFA production in a rapidly growing market [22].

The transformation of novel non-model microalgae species is a multi-dimensional puzzle with several unknowns. The successful transfer of the DNA into the algal cell without causing lethal damage, the sufficient expression of a suitable selection marker and the efficient recovery of the transformed cells are all essential for achieving adequate transformation efficiency. This had been achieve in several representatives of heterokont algae [27] [28]. Green algae appear to be more difficult to transform, and until recently, *C. reinhardtii* remained the only green algal species accessible to metabolic engineering. Recently many protocols for transformation of other green algae species have been presented [27]–[35], but efficient metabolic engineering remains to be confirmed.

Based on our experience with transformation of *L. incisa*, we think that success depends on consideration of all of the following issues:

- A suitable combination of promoter and selection marker must be established. Our initial attempts with plasmid pSP124 designed for *C. reinhardtii* transformation [35] were unsuccessful in *L. incisa*. Selecting the endogenous *L. incisa RBCS* promoter and testing it in *C. reinhardtii* allowed us to determine its optimal length for driving transformation, which proved effective in *L. incisa*. Our results open the door to further improvement. For example, one could try to introduce sequence elements that might insulate the promoter from negative effects from the surrounding context, as was achieved with the *HSP70A-RBCS2* combination in *C. reinhardtii* [36]. Introduction of an intron might also improve transformation yield, as shown for *C. reinhardtii* [35]. Other resistance markers also need to be tested.
- The clumping behavior of *L. incisa* represented a major challenge in developing adequate transformation and gene transfer technologies. Finally, the cultivation of the strain in a rich organic medium, accompanied by mild mechanical treatment, was successful in producing cultures composed of predominantly single cells, adequate for efficient transformation.
- Electroporation, at carefully optimized conditions, was found to be the most effective method of gene transfer, allowing the recovery of transformed cells at an adequate frequency. But we had no success with particle gun bombardment. Electroporation has the significant advantage of requiring only one plating step following transformation, when using antibiotics selection, whereas particle gun bombardment requires replating on selective medium. In *Haematococcus pluvialis*, we estimate that only five percent of cells survive the plating steps required during the bombardment, recovery and replating procedures even before the selection for transformants becomes effective (S. Boussiba, unpublished results). Usefulness of bombardment may still be explored with non-lethal antibiotics like spectinomycin [37], or complementation approaches, where no replating is necessary.
- The adequate expression of heterologous marker genes is an additional and final obstacle for achieving satisfactory transformation efficiency, mostly due to different codon usage in different organisms. The codon usage of the *ble* gene is quite similar to that in *L. incisa*. However, it is generally accepted that the highest expression rate of transgenes in recombinant microalgae is achieved with constructs containing endogenous selection markers as compared to the heterologous genes. This is why the development of selection markers, based on endogenous genes, could be an important part of an efficient “molecular toolbox” and an essential step in the future.

In summary, we have managed to demonstrate the successful stable transformation of *L. incisa* utilizing a partial set of possible optimization steps available. Moreover, the usage of the transformation method described above will lead to the development of a complete molecular toolbox, including endogenous markers and sets of constitutive and inducible promoters. We have furthermore demonstrated successful metabolic engineering in this alga by restoring AA biosynthesis to the mutant P127, via transformation of the wild-type *DES5* gene with its own promoter linked to the *ble* selection marker. The approach developed in this work may now permit the engineering of the LC-PUFA metabolism in *L. incisa* by expression of homologous and heterologous desaturases and elongases for the production of high value ω-3 LC-PUFA of commercial interest.
